# Tracing the Flow of Perceptual Features in an Algorithmic Brain Network

**DOI:** 10.1038/srep17681

**Published:** 2015-12-04

**Authors:** Robin A. A. Ince, Nicola J. van Rijsbergen, Gregor Thut, Guillaume A. Rousselet, Joachim Gross, Stefano Panzeri, Philippe G. Schyns

**Affiliations:** 1 Institute of Neuroscience and Psychology, University of Glasgow, Glasgow, G12 8QB, UK; 2 Neural Computation Laboratory, Istituto Italiano di Tecnologia, Rovereto, 38068, Italy

## Abstract

The model of the brain as an information processing machine is a profound hypothesis in which neuroscience, psychology and theory of computation are now deeply rooted. Modern neuroscience aims to model the brain as a network of densely interconnected functional nodes. However, to model the dynamic information processing mechanisms of perception and cognition, it is imperative to understand brain networks at an algorithmic level–i.e. as the information flow that network nodes code and communicate. Here, using innovative methods (Directed Feature Information), we reconstructed examples of possible algorithmic brain networks that code and communicate the specific features underlying two distinct perceptions of the same ambiguous picture. In each observer, we identified a network architecture comprising one occipito-temporal hub where the features underlying both perceptual decisions dynamically converge. Our focus on detailed information flow represents an important step towards a new brain algorithmics to model the mechanisms of perception and cognition.

Since the seminal work of Turing^1^, most influential models in cognitive neuroscience are implicitly cast in terms of information processing^2^ in a densely connected, hierarchically organized network^3^ with bottom-up and top-down flows of information^4^,^5^. To illustrate, consider that in predictive coding, a prediction implies explicit knowledge of *what* specific information is propagated down the visual hierarchy^4^,^6^. Likewise, a decision implies the successful match of sequentially accrued, sensorily coded information (the evidence) with memorized information^7^ (categorical knowledge) serving as decision criterion^8^,^9^. Although existing models of prediction, memory, sensory coding and decision^10^,^11^ are all implicitly cast within an information processing framework, reverse engineering the flow of this specific information from brain data remains a considerable challenge. To address this, we develop and apply new methods that identify the neuronal processes or communication pathways with specific perceptual/cognitive information processing roles.

## Results

**Figure 1:**
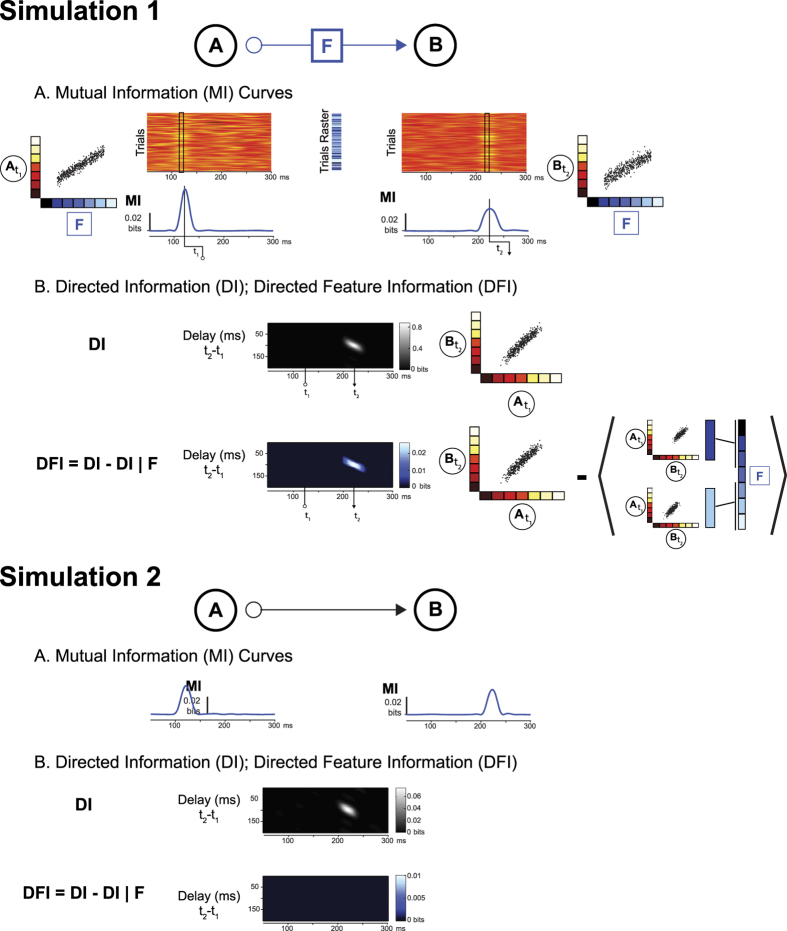
Simulation 1. *Simulation of Directed Information (DI) and Directed Feature Information (DFI).* With DFI, we can establish that network node A codes and sends feature F at time t_1_ to B, that receives and codes F at time t_2_. (**A**) Mutual Information (MI) Curves. We simulated MEG responses from two nodes (see Methods), such that the values of stimulus feature F (e.g. the graded presence of the left eye in the face stimulus, represented with a raster of shades of blue) modulates the activity of node A at time t_1_, which in turn modulates the activity of node B at time t_2_ (node B was not directly modulated by F). The red raster plots illustrate the simulated single-trial responses of nodes A and B to values of stimulus feature F. The left and right scatters illustrate the relationship existing at time t_1_ (and t_2_) between the single-trial activity of node A (and node B, color-coded with a red raster) and the values of stimulus feature F (color-coded with a blue raster). Mutual Information time courses quantify this relationship for each time point. (**B**) Directed Information (DI); Directed Feature Information (DFI). For different delays, DI quantifies the total causal transfer of signal information from node A to node B-i.e. A sends MEG signal information between 100 to 140 ms to B which receives it between 210 to 230 ms–i.e. with a delay of 90 to 110 ms matching the parameters of the simulation. We schematize this relationship between sources A and B at different time points with scatters. DFI goes further by conditioning DI on the variations of feature F (represented with the conditioning performed on the different values of F, represented as different shades of blue) and so DFI quantifies how much of the total DI transfer is *about* feature F. **Simulation 2** *Simulation of Directed Information (DI) with no Directed Feature Information (DFI).* We simulated MEG responses from two nodes (see Methods), such that the stimulus value independently modulated the activity of node A at time t_1_, and node B at time t_2_. The activity of B at time t_2_ was further modulated by the baseline activity of A at t_1_. MI curves, and DI and DFI transfers are shown as Simulation 1. Here, there is DI corresponding the information peaks in each node, but there is no positive DFI. In this case the content of the communication is not related to the stimulus feature, it reflects instead the background activity at source A. These two functionally distinct situations cannot be dissociated based on DI and MI alone, but DFI allows us to correctly separate them.

The conceptual framework underpinning these results is encapsulated in Simulation 1 Fig. 1, where network node A communicates stimulus feature F to network node B (see blue arrow). To build information processing models of perception and cognition, we require a level of functional understanding with at least this level of specificity: “Node A codes stimulus feature F (e.g. at t_1_) and then sends it to node B (e.g. which receives it at e.g. t_2_).” The Directed Feature Information (DFI) measure we develop and apply to perceptual decisions provides this level of specificity.

To illustrate with a simulated example, suppose that variable F measures the graded presence of any feature of interest in the stimulus—e.g. the ‘trials raster’ in Fig. 1 illustrates with shades of blue the graded presence of the left eye across trials. On each trial, we simulate presenting a stimulus (represented as a value of F, a shade of blue) and measure the response of two simulated brain network sources A and B (see Methods, Network Source Simulation). We then use Mutual Information (MI) to quantify at each time point the strength of the relationship between the graded variable F and corresponding simulated Magnetoencephalographic (MEG) responses of A and B (see Fig. 1, Simulation 1, Panel A). We find (see adjacent blue MI curves) that node A codes feature F with a peak at t_1_, whereas node B codes feature F with a peak at t_2_. We then ask the question: Does A causally send the information about feature F available at t_1_ to B that receives it at t_2_?

Before we answer the question, first consider the shortcoming of state-of-the-art methods (see Discussion for a more thorough discussion). In Fig. 1, Simulation 1, Panel B, the grey matrix illustrates the Directed Information (DI, often called Transfer Entropy (TE), see Methods for definition) between the single trial activities of nodes A and B, suggesting a relationship between the activations of node A at t_1_ and the activations of B at t_2_ (see adjacent scatter) for this lag. We can conclude that the activity at node A exerts a causal influence on the activity at node B and reconstruct a communication network on the basis of such causal relationships between pairs of nodes. However, whereas such methods establish that the two network nodes communicate, they do not specify what the nodes are communicating about. That is, we do not know which specific stimulus feature, if any, A sends to B.

Our contribution to the field of brain connectivity is Directed Feature Information (DFI), a novel measure that quantifies the causal transfer of specific information content, here a stimulus feature F. Briefly stated, the DFI of stimulus feature F from node A to B is defined as:


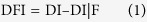


where DI is the total directed information transferred from A to B (i.e. any predictive information, possibly including stimulus features) and DI|F is the DI conditioned on a specific feature F (e.g. presence/absence of the left nuns’ face, see scatter plots illustrating Equation 1 in Fig. 1, Simulation 1, Panel B). As Fig. 1 illustrates, conditioning on F has the effect of computing DI as if the value of F was fixed (and then averaging DI across these different fixed values). DI|F measures the average communication that occurs independently of the sampling variations of F. Subtracting DI|F from the overall DI, we obtain a measure of the transfer that is directly related to the sampling variations of F. Positive DFI therefore quantifies the transfer *about* stimulus feature F from node A to B (see Methods for details).

Informally, node A can transfer different sorts of information to B. For example, computer network nodes exchange synchronization information related to opening and closing communication channels, in addition to sending an email from A to B. DI from A to B would measure the transfer of all such information in the brain. But our aim is to identify the transfer of perceptual content (i.e. an email in the analogy). Thus, we recompute DI, but disabling the effect of feature F on DI (i.e. DI|F). DI|F measures the transfer of all information excluding information about the targeted feature. Thus, the difference DI–DI|F quantifies the transfer that is about feature F.

In Fig. 1, Simulation 1, Panel B DFI (blue matrix) illustrates that about 3.5% of the DI communication between nodes A and B is about feature F—i.e. the ratio .029 (DFI)/0.82 (DI) = 0.035. From DFI, we can reconstruct the network of the nodes that code and transfer a specific stimulus feature. Thus, with DFI we can in principle reconstruct the information processing networks subtending perceptual and cognitive tasks. Note that DFI networks necessarily subtend the DI network because by definition DFI is less than DI. Further note that DFI requires coding of F in both the sending and receiving source (see Simulation 1 of Fig. 1), though such coding does not necessarily imply DFI (see Simulation 2 of Fig. 1, which shows a case where there is coding and communication, but the communication is not about the stimulus feature that is coded).

**Figure 2:**
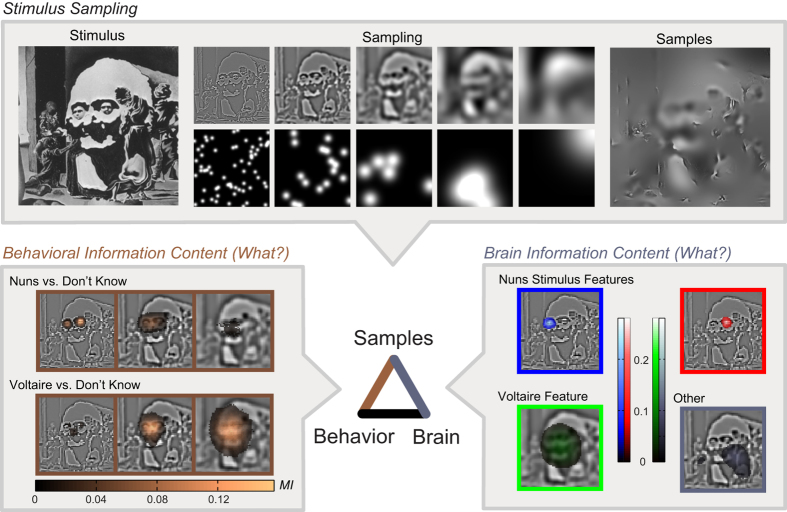
What Information? Stimulus Sampling. The original ambiguous picture is split into five non-overlapping Spatial Frequency (SF) bands of one octave each. Each band is sampled with randomly positioned Gaussian apertures of contiguous pixels. Samples are combined to produce a different random sampled image on each experimental trial. Behavioral Information Content. Mutual Information (MI) between single-trials stimulus samples (independently for each pixel and SF band) and corresponding behavioral responses for Observer 1. Significant (*p* = 0.01) MI is color-coded and overlaid onto the original SF bands for visualization. Brain Information Content. Stimulus features derived from a MI analysis between single-trial stimulus samples and corresponding MEG source activities, followed by a dimensionality reduction. Four example stimulus features that are represented in the brain are shown (chosen from the 21 available for this observer). Three of these (labeled with Red, Green and Blue) overlap with behavior for this observer, one (Grey) is disjoint from behavior. The section of the Dali painting “Slave Market with a Disappearing Bust of Voltaire” is © Salvador Dalí, Fundació Gala-Salvador Dalí, VEGAP, 2014 and is excluded from the Creative Commons license covering the rest of this work.

We applied our information processing framework to the task of perceiving either *the Nuns, Voltaire*, or *don’t know* from information samples randomly extracted with Bubbles^12^ from Dali’s ambiguous painting named *Slave Market with Disappearing Bust of Voltaire*^13^. We know from psychophysics that individual observers will attend to and use specific information content from the stimulus to perceive^13^ or categorize it^14^,^15^. We therefore aimed to first identify the information underlying the perception of each observer and then characterize the brain network that dynamically processes this information. To achieve these aims, on each trial we randomly sampled contiguous pixels, with small Gaussian apertures distributed across 5 non-overlapping Spatial Frequency (SF) bands of one octave each, as illustrated in Fig. 2 (see Methods). Each stimulus was thus a sparse version of the original painting. The task of each observer was to perceptually decide (i.e. explicitly report with response keys) whether they saw the *nuns, Voltaire* or neither (“*don’t know*”) from the samples present in each stimulus. We expected the *nuns* to reside primarily in High SF and *Voltaire* in Low SF^13^. We concurrently measured each observer’s brain activity with MEG (see Fig. 2 and Methods). Behavioral results are shown in Methods.

First, we identified the stimulus information content associated with each behavioral perceptual decision (gold link and “Behavioral Information Content” box in Fig. 2). Specifically, across trials we quantified the association (Mutual Information^16^, MI) between single-trial stimulus samples (independently for each pixel and SF band) and the corresponding behavioral responses (see Methods). The behavioral information images reveal that Observer 1 perceived the *Nuns* with regions representing the two nuns’ faces in High-SFs and *Voltaire* with Lower-SF regions representing his face (highlighted in gold and presented for significant pixel clusters in SF bands 1 to 3). This implies that this observer’s brain network uses an algorithm that must process at least this content to perceive the *Nuns* and *Voltaire*.

To reveal the nodes of the brain network, we applied Independent Component Analysis^17^ (ICA) and decomposed the multi-channel MEG brain signal into the main sources of activity (*network nodes*, see Methods). We then identified (with MI and Non-negative Matrix Factorization^18^, NMF) a set of stimulus features that modulate the activity of these nodes post stimulus onset (see Methods). In Fig. 2, the Brain Information Content box represents with a color-code the main stimulus features coded in the brain of Observer 1, revealing that some of these overlap with behavioral content (e.g. compare the Blue nun’s face feature and also the Red and Green features with those in the Behavioral Information Content box).

**Figure 3:**
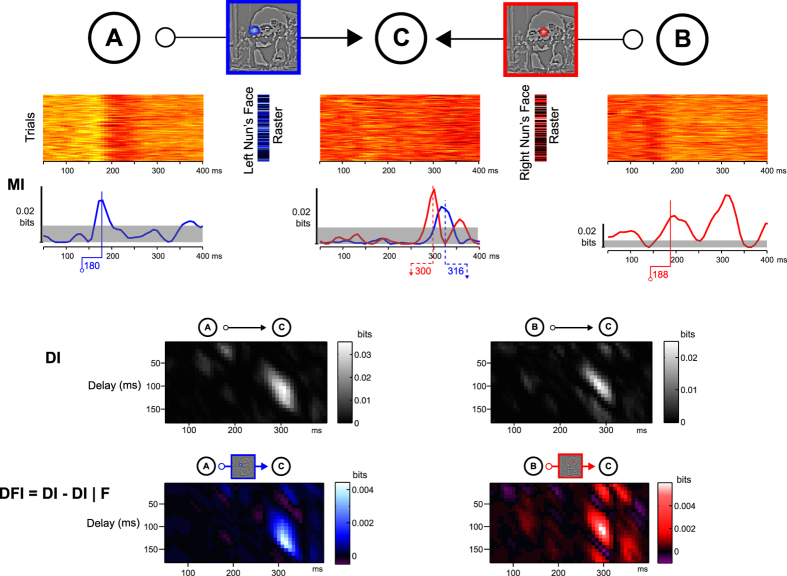
Directed Information (DI) and Directed Feature Information (DFI). Having identified in Fig. 2 *What* features the brain of Observer 1 processes for perceptual decisions (framed in blue and red in Fig. 2 and here), with DFI we illustrate coding and transfer of these features between right (node A) and left (node B) occipito-temporal regions and inferior parietal region (node C). MI. Color-coded Mutual Information times courses for each feature (single-trial left and right nun’s faces values, see corresponding color-coded rasters) and network node (single-trial MEG activity). DI. Directed Information reveals that A and B send MEG signal information to C with a ~120 ms of delay. DFI. Directed Feature Information reveals the transfer that is about each feature, reconstructing two links of an information processing network of three nodes. The section of the Dali painting “Slave Market with a Disappearing Bust of Voltaire” is © Salvador Dalí, Fundació Gala-Salvador Dalí, VEGAP, 2014 and is excluded from the Creative Commons license covering the rest of this work.

With DFI we can now characterize *Where, When* and *How* this behaviorally relevant information content flows in brain networks. Figure 3 illustrates the coding and transfer of stimulus features across three network nodes selected as examples from the experimental data set. Specifically, from the MI plots, we note that a right occipito-temporal (OTR) node A and a left OTL node B code the left (coded in blue) and right (coded in red) nuns faces respectively, with peaks at 180 and 188 ms. We also note that inferior parietal source C codes both features, with peaks around 300 ms. We ask whether A and B send the feature they code to C. With DI, we first establish that A communicates to C, and B communicates to C. With DFI, we then show that a proportion of the communications from A to C (vs. from B to C) is about the left (vs. right) nuns face.

**Figure 4:**
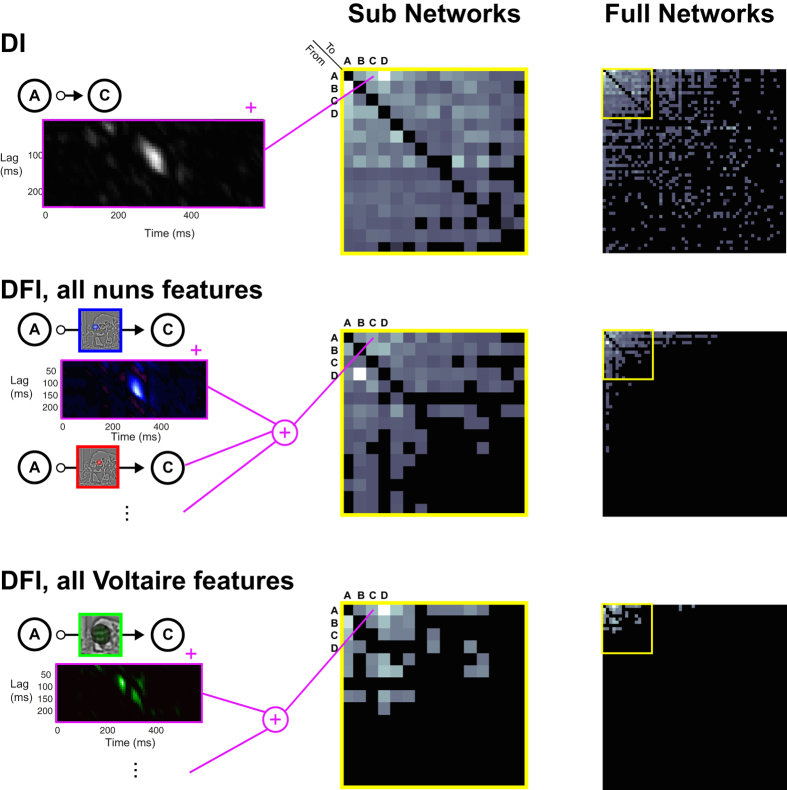
DI and DFI Communication Networks. The Full Networks and Sub Networks matrices represent the DI and DFI communication networks of Observer 1. In all matrices, nodes are ordered by total feature degree (see Methods). For references, the (**A–C**) nodes of Fig. 3 and their pairwise communications are labelled. Each cell in the matrices reports a directed (i.e. non-symmetric) and integrated (between 50–350 ms post-stimulus and across 8–240 ms delays) measure of communication. The Full Networks matrices report the communications between all node pairs. The yellow framed Sub Networks matrices expand sections from the Full Networks matrices. DFI matrices are independently constructed for the communication of the features subtending each perception (i.e. *Nuns* and *Voltaire*) of the ambiguous stimulus. The section of the Dali painting “Slave Market with a Disappearing Bust of Voltaire” is © Salvador Dalí, Fundació Gala-Salvador Dalí, VEGAP, 2014 and is excluded from the Creative Commons license covering the rest of this work.

For each observer, we repeat computations of DI and DFI across all identified node pairs, sum DI values (between 50–350 ms post stimulus, and for 8–240 ms delays) and report these in a DI network of overall communications (see Fig. 4, Full Networks). We also sum DFI communications of all nuns (vs. Voltaire) features required for perceptual decisions and report these networks of perception specific feature communications (see Methods and Fig. 4, Full Networks). Note that DFI networks (highlighted in yellow) differ across perceptions and have a sparser structure than the corresponding DI network. For the illustrated Observer 1, only 20% of all significant DI connections comprise connections with significant DFI (for other observers, mean = 5.3%, range = 3.2–8.7%, see Supporting Figures S1, S2, S3, S4). Figure 4 shows all three networks for Observer 1 at both zoom levels (yellow insert) to allow a direct visual comparison. We quantify this difference with the ‘average degree’ of the 60-node network (the average number of significant connections per node, see Methods). For the illustrated Observer 1, the average degrees were 3.1, 1.87 and 0.7 for the DI, nuns DFI and Voltaire DFI networks respectively. Across all 5 observers, the difference in average degree between the DI (3.1) and the nuns DFI (0.7) was significant, as was the difference between the DI and the Voltaire DFI (0.7) networks (one tail sign-rank test, both p = 0.03). This demonstrates that networks constructed based on the communication of the specific information content underlying each perception (i.e. DFI) are both distinct from each other, as well as strikingly different from networks obtained with conventional measures of functional connectivity (here, DI).

**Figure 5:**
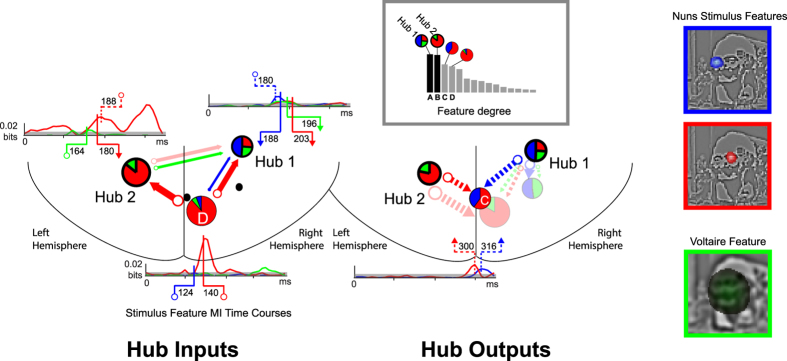
Algorithmic Brain Network. Gray Insert. DFI node total feature degree reveals network hubs (gray frame, total number of significant inward and outward DFI connections, summed over features, for each node). Hub Inputs. On the line-drawn back of the brain with labeled left and right hemispheres, network schematics show the detailed features and timing properties for sections of the feature networks; specifically connections into the hub nodes. We represent DFI with color-coded arrows that denote the transfer of the color-framed stimulus features (reproduced from Fig. 2) underlying both perceptions in Observer 1. Specific examples illustrated with time course plots are shown with full saturation. Pie charts in the network schematics summarize proportional coding of stimulus features at each node. In the left schematics, solid arrows illustrate transfer into the hubs. Adjacent color-coded time courses plot the association (MI) between stimulus feature and network node activity; shaded grey region indicates threshold of statistical significance (p = 0.01, multiple comparison corrected over ICA sources, time points and component filters). White circle labels indicate sending times of highlighted DFI; arrowhead labels receiving time. Hub Outputs. The schematics illustrate the coding and transfer (with dashed arrows) of color-coded DFI out of the hubs. The section of the Dali painting “Slave Market with a Disappearing Bust of Voltaire” is © Salvador Dalí, Fundació Gala-Salvador Dalí, VEGAP, 2014 and is excluded from the Creative Commons license covering the rest of this work.

In Fig. 5, we examine the specifics of the *where, when* and *how* of feature processing and transfer in the DFI networks, highlighting four nodes represented with color-coded pie charts, with best fit dipole source locations represented on a schematic brain (horizontal plane, see Methods). These nodes communicate most perception-relevant features (see grey-level histograms of feature transfer degree, the sum across stimulus features of the number of significant incoming and outgoing DFI links^19^, and Methods). Of these, two nodes (Hub 1, Hub 2) covering the main occipito-temporal regions act as functional hubs–i.e. they receive and send the highest number of distinct stimulus features (we represent hubs with black bars on the histogram and with thick bordered pie charts). We now illustrate the detailed communications into (left schematic, plain arrows) and out of (right schematic, dashed arrows) the hubs with two further nodes (indicated with thin line pie charts) that most strongly communicate the Red, Green and Blue (RGB) framed stimulus features underlying perceptual decisions (reported from Fig. 2 for clarity of exposition).

Having addressed *where* in the network the main feature communications occur, we now address *when* and *how*, starting with the left *Hub Inputs* network schematic (Fig. 5). Pie charts summarize the coding of the correspondingly colored stimulus features in individual network nodes. Adjacent colored curves represent their coding dynamics. Starting with the early visual cortex node (marked with a “D”), its pie chart quantifies in red the early dynamic coding of the right nun’s face (see red stimulus feature MI time course curve underneath). This node then sends this feature to the left Hub 2, as the red arrow indicates. It also sends the left nun’s face (coded in blue) to the right Hub 1, as the blue arrow indicates. Arrow thickness represents the strength of feature transfer. Throughout the panel, hollow circles denote “node sending” and arrowheads denote “node receiving.” To illustrate, a plain red hollow marker at 140 ms on the time course of node D indicates when it sends the right nun’s face to Hub 2. The plain red arrow marker on Hub 2 indicates that the node receives the right nun’s face at 180 ms.

Interestingly, Hub 1 receives and then sends the feature content of each perceptual decision. Here, the plain blue, red and green arrow markers on the adjacent time courses indicate that Hub 1 receives the contra-lateral left nun’s face first (coded in blue, at 188 ms, sent from node A as just explained), followed by the face of Voltaire (coded in green, at 196 ms, sent from the left hub at 164 ms), followed finally by the ipsi-lateral right nun’s face (coded in red, at 203 ms, sent from node D again). But the flow of information content does not stop in the hubs. Hubs 1 and 2 also gate and send information content (dashed arrows represent sending in the right *Hub Outputs* network schematics (Fig. 5), where the discussion of the results now moves to). To illustrate, Hub 2 sends the right nun’s face at 188 ms and the Hub 1 sends the left nun’s face at 180 ms. In both cases, the same inferior parietal node (labeled C) receives the right and left nuns’ faces at 300 and 316 ms, respectively, and did not code any of these features before, as indicated in the adjacent red and blue time courses (see right panel of the Fig. 5; this is the network segment illustrated in detail in Fig. 3). The hubs also send other features to others sources (represented with transparent arrows and nodes) but we will not detail them here.

**Figure 6:**
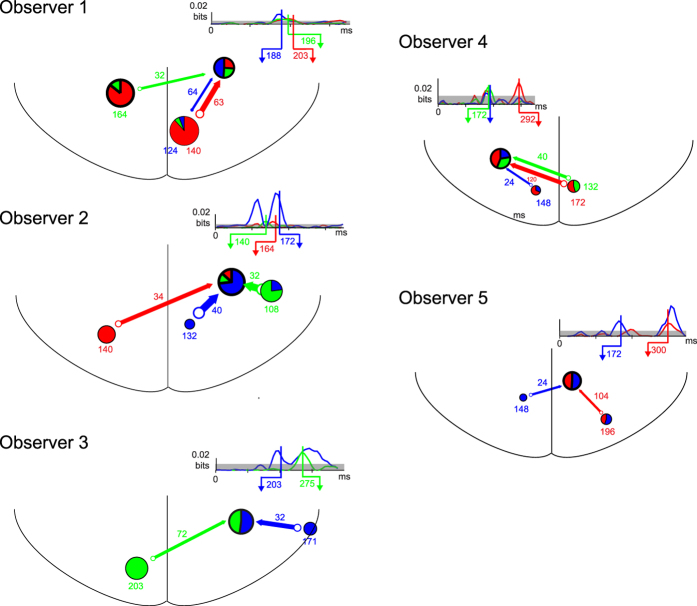
Common Feedforward Architecture. DFI network schematics represent DFI with color-coded arrows that denote the transfer of the color-framed stimulus features underlying both perceptions in each observer (from Fig. 5, and Supplemental Figures 2–5). Pie charts in the network schematics summarize proportional coding of stimulus features at each node, where blue and red color-code the left and right nuns’ face, respectively, whereas green codes the more idiosyncratic, observer-specific Voltaire features. Color-coded time courses adjacent to the occipito-temporal hubs plot the association (MI) between stimulus feature and network node activity; shaded grey region indicates threshold of statistical significance (p = 0.01, multiple comparison corrected over ICA sources, time points and component filters). Arrowhead labels indicate the receiving time of highlighted DFI. Color-coded numbers near each node indicate the sending time of the features and color-coded numbers near each arrow indicate the corresponding transfer lag to the hub.

Figure 6 shows the *Hub Input* network details for all five observers (see Fig. 5 and Supplemental Figures S1, S2, S3, S4, for complete results). These segments of reconstructed networks show consistency in the spatio-temporal information processing architecture they reveal. An early time window (spanning, across observers, 110 to 200 ms following stimulus onset) reflects initial coding of the features underlying perceptual decisions of *the nuns* (in red and blue) and *Voltaire* (in green). This coding occurs as expected in multiple (retinotopic) occipital regions, showing in a majority of observers a strong property of contra-laterality of coding (e.g. the left nuns face in blue or the right nuns face in red initially coded in the right or left hemisphere, respectively). Following initial coding, the features underlying both perceptions converge onto a common lateral hub, located in 4/5 observers in the right occipito-temporal region (left in one observer) of the ventral stream. In this unique region of convergence, all features are coded in a subsequent time window spanning 140 to 300 ms post-stimulus. From the hubs, features are then dispatched to other regions, including inferior parietal regions in 4/5 observers. Supplemental Figure S6 shows the topologies of the ICA sources and their 3D localization obtained using a dipole fitting procedure (see Methods) for the hubs of each observer. To determine whether the topologies for the functionally identified hub sources are more similar across observers than randomly selected sources, we performed a permutation analysis (see Methods). The median percentile of all hub-hub correlations across observers was 99% of all random cross-observer source correlations. A rank-sum test for a difference in median of the raw correlation value for hub-hub cross-observer comparisons vs. random cross-observer comparisons was highly significant (rank sum test; p = 1.4 × 10^−5^).

The dynamic flow of information processing just described is algorithmic in the sense that it specifies what specific stimulus features subtend perceptual decisions and then characterizes *where, when* and *how* network nodes represent and transfer each feature. To be clear, the novelty of our results is not to demonstrate again the global flow of information from visual cortex to the ventral stream, as this is well established^20^ and flow can be demonstrated using as stimuli full images of faces, objects or scenes^21^. Rather, we reconstructed the flow of the specific visual features that underlie perceptual and cognitive decisions, specifying the effective visual information and its early processing in brain networks. The finding that contra-laterally coded perceptual features (for both perceptions) originating from different occipital regions converge on a unique occipito-temporal hub constrains the functional interpretation of network nodes and the pathways of feature communication relevant for perceptual decision (i.e. behavior).

## Discussion

Our approach is based upon the notion that the brain is an organ of information processing. Yet, to our knowledge, no method existed to characterize brain networks by the flow of the specific information content that nodes dynamically receive, code, gate and send during a perceptual or cognitive task. Whereas an emphasis on the information content or representation of neural responses has been recently gaining popularity^10^,^22^,^23^,^24^, and a network view of brain function based on various connectivity measures is now well established^3^,^25^,^26^, to date these two viewpoints have not yet been combined. Dynamic Causal Modelling^27^,^28^ fits circuit models to data and as such can in principle reveal important aspects of information flow across nodes of networks, including stimulus dependent couplings. Yet, in its present form it does not reveal which stimulus features are communicated and which ones are not, and it suffers from the potential drawbacks of model-based approaches. The primary such drawback is a dependence on the correctness of model assumptions, which may limit the generality of application and complicates exploratory analyses. Thus our model-free estimation of stimulus information transfer complements previous approaches and offers a new general framework that represents brain networks at an algorithmic level.

Key to the framework is our development of DFI, a measure of directed and causal transfer of specific information content (i.e. stimulus features) between network nodes. This methodological development provides an as yet unparalleled level of analysis for coding and transfer of feature content in a brain network. We validated our approach in a simulation and on an ambiguous visual stimulus sampled with Bubbles. The resulting algorithmic networks demonstrate surprising and novel information processing properties warranting further investigations.

## Implications for Task-Relevant Visual Information Processing

First, our method distinguishes stimulus features relevant for perceptual decisions from those that do not affect decision but are nevertheless reliably coded in the neural signal (highlighted in grey, in the Brain Information Content of Fig. 2). This duality not only demonstrates that the brain codes more information than is strictly necessary for overt behavior. It also suggests a methodology for understanding the network processing of task-irrelevant vs. task-relevant information in brain networks with profound implications for prediction, attention and decision making. To illustrate task-relevant processing, consider the following point and its implications: Though the Behavioral Information Content box reveals that two nun’s faces underlie perceptual decisions of *the Nuns*, the Brain Information Content box shows that the information representation in MEG sources (computed independently of behavioral responses) consists of each face as a separate stimulus feature. Furthermore, Fig. 5 (see also Supplementary Figure S1-S4) illustrates that a different, contra-lateral brain region (Hub 1 and 2) processes each nun’s face around 200 ms, before both faces converge to a more central region 300 ms post stimulus. Not only does this reveal which region processes what content in a distributed network structure, it also constrains the pathways and timing of communications of perception-relevant features in the brain’s algorithms. Considering this timing, note that the hubs receive and send information about *both* perceptions, within time windows compatible with the timing and source localisations of the N/M 170 and P300 evoked activity, which are known to be involved in the coding of face features^22^,^29^,^30^ and decisions on the basis of these features^31^,^32^, respectively. Note that both perceptions include faces, at different spatial locations and scales (small HSF faces in each visual hemifield for the nuns and a larger central LSF face for Voltaire). It is worth noting that all these are sent as features to the hub of each observer, with a spatio-temporal localization congruent with evidence for typically right-lateralized face processing in EEG, MEG and fMRI^29^,^33^,^34^,^35^,^36^,^37^. The information hubs receive can be either ipsi- or contra-lateral to the hub, suggesting that distinct, anatomically specialized communication channels converge on the hub, sending complementary information (e.g. contra- and ipsi-lateral stimulus information such as the nuns’ faces). Together, these findings suggest the development of more sophisticated, time-resolved algorithmic networks to model the information processing mechanisms of perception and cognition.

## Implications for Functional Interpretation of Brain Network Nodes

Our findings also constrain the functional interpretation of the nodes. For example, the fact that the left and right hubs receive and dispatch perception-relevant features suggests that they might gate (passing and/or blocking) visual information for subsequent perceptual decisions. The fact that the inferior parietal node receives both nuns faces around 300 ms from the hubs suggests that it could be a primary candidate to examine feature integration for perceptual decision^38^. Interpretation of the precise computational functions of network nodes would be enhanced with a clearer understanding of how the information each node sends differs from the information it receives.

## Extensions to Other Visual Inputs, Sensory Modalities and Brain Measures

Our framework suggests promising avenues for a wide range of applications in neuroscience. Starting with sensory modalities, our methods can in principle generalize to any of them, provided that an appropriate stimulus sampling strategy is designed to match the considered problem in the modality in question. For example, in the auditory domain, stimuli could be sampled over a set of suitable spectro-temporal stimulus dimensions, using a strategy similar to bubbles^39^. Similarly, more sophisticated sampling schemes can be designed for direct sampling of higher-level features via generative models^40^ (e.g. random sampling of time varying facial muscle groups^41^,^42^, or a frequency-specific tagging of stimulus components to reconstruct the networks involved in their higher-order integration into e.g. a face, object or scene). Higher-level features could explicitly account for various forms of invariance, allowing network reconstruction of more abstract feature processing and transfer than regions of contiguous pixels. The approach is also straightforwardly generalizable to other stimulus categories and tasks, such as different categorizations of faces, objects and scenes. Furthermore, we can also inspect the information flow for other features that are not relevant for behavior. In fact, Fig. 2 (Brain Information Content, Other, Grey Box) reveals a stimulus feature that is not obviously relevant for perceptual decision but nevertheless coded in the brain. We could track the coding, transfer and suppression of such task-irrelevant features between stimulus onset and response. Generally speaking, we could perform an analysis that uses MI to rank each feature for its relative contribution to perceptual or categorical decisions (in different categorizations of the same stimuli). And we could independently examine which of these features are coded in the brain (with what strength), transferred, suppressed and integrated between stimulus onset and behavior. In all future applications, the main challenge remains the careful design of a sampling strategy; all other elements of the framework should be applicable with minimal modification.

Considering neural responses, our methods can be applied to brain signals from any neuroimaging or electrophysiological recording modality. For example, high-field fMRI would allow finer spatial resolution of network node locations. Recordings from implanted electrode arrays could provide higher temporal and spatial granularity, from signals with different levels of integration such as neuronal spikes and Local Field Potentials^43^. Networks could be constructed using recordings from distal sites or on a more local scale from high-density arrays. Across measurement modalities (or levels of granularity, or species), our information theoretic methods allow direct quantitative comparisons of information processing within brain networks.

One assumption of our approach is that when a stimulus changes (via the information sampled with Bubbles) only stimulus-related processing changes. While probably true, it is possible that stimulus changes will also elicit the use of different networks, or use of different network modes. For example, we have shown that different stimulus features could be coded in the phase of different oscillatory bands (e.g. the mouth of a face in theta [4–8 Hz] oscillations vs. the eyes in beta [12–24 Hz] oscillations^44^). Though not explicitly tested here, it could be that different input features elicit different oscillatory networks, or that the same network could enter different modes (slow vs. fast oscillations) as a function of e.g. the scale of the features. Such questions will be the object of further investigations.

These techniques can be applied to a wide-range of tasks and manipulations in healthy and clinical subjects, which we anticipate will provide significant insights that would be impossible to obtain without such a consideration of functional information processing. All of this potential can be addressed because we are now finally able to frame the brain as an algorithmic mechanism that processes information content.

## Methods

### Observers

Five observers with normal (or corrected to normal) vision participated in the experiment, which was conducted in accordance with approved guidelines. We obtained informed consent from all and ethical approval from the Glasgow University Faculty of Information and Mathematical Sciences Ethics Committee.

### Experimental Protocol

We cropped the ambiguous portion of Dali’s *Slave Market with the Disappearing Bust of Voltaire* to retain the bust of Voltaire and the two nuns. The image was presented at 5.72° × 5.72° of visual angle on a projector screen (image size was 256 × 256 pixels)^13^. We sampled information from the cropped image using *bubble masks*^12^ made of randomly placed Gaussian apertures to create a different sparse stimulus for each trial (see Fig. 2). We decomposed the Dali image into five independent *Spatial Frequency* (SF) bands of one octave each, with cutoffs at 128 (22.4), 64 (11.2), 32 (5.6), 16 (2.8), 8 (1.4), 4 (0.7) cycles per image (cycles per degree of visual angle). For each SF band, a bubble mask was generated from a number of randomly located Gaussian apertures (the bubbles), with a standard deviation of 0.13, 0.27, 0.54, 1.08, and 2.15 degrees respectively. We sampled the image content of each SF band by multiplying the bubble masks and underlying greyscale pixels at that SF band, and summed the resulting pixel values across SFs to generate the actual stimulus image. The stimulus remained on the screen until the observer depressed one of three possible response keys, according to which aspect of the image they perceived: “the nuns” (N), “Voltaire” (V), or “don’t know” (DK). A fixation cross was presented from 500 ms prior and until stimulus onset and observers were instructed to maintain fixation during each trial. The total number of Gaussian apertures remained constant throughout the task, ensuring that equivalent amounts of visual information was presented on each trial, at a level (60 bubbles) found previously to maintain “don’t know” responses at 25% of the total number of responses^30^. Since the underlying image was always the same, all analysis was performed on the bubble masks controlling visibility. For analysis, we down-sampled (bilinear interpolation) the bubble masks to a resolution of 64 × 64 pixels.

### Behavioral response data

Tables 1 and 2 provide full behavioral response data for all five observers.

## MEG data acquisition and preprocessing

Stimuli were presented in runs of 150 trials, with randomized inter-trial intervals of 1.5–3.5 s (mean 2 s). Observers performed 4–5 runs in a single day session with short breaks between runs. Observers completed the experiment over 4–5 days. We measured the observers’ MEG activity with a 248-magnetometer whole-head system (MAGNES 3600 WH, 4-D Neuroimaging) at a 508 Hz sampling rate. We performed analysis with the FieldTrip toolbox^45^ and in-house MATLAB code, according to recommended guidelines^46^. For each observer, we discarded runs based on outlying gradiometer positions in head-space coordinates. To do this, we computed the Mahalonobis distance of each sensor position on each run from the distribution of positions of that sensor across all other runs. Runs with high average Malahanobis distance were considered outliers and removed. The distances were then computed again and the selection procedure was repeated until there were no outlier runs (Mahalonobis distances >20) . We high-passed filtered data at 1 Hz (4^th^ order two pass Butterworth IIR filter), filtered for line noise (notch filter in frequency space) and de-noised via a PCA projection of the reference channels. We identified noisy channels, jumps and other signal artifacts using a combination of automated techniques and visual inspection. We then epoched the resulting data set (mean trials per observer 3396, range 2885–4154) into trial windows (−0.8 s to 0.8 s around stimulus onset) and decomposed using ICA^17^, separately for each observer. We identified and projected out of the data the ICA sources corresponding to artifacts (eye movements, heartbeat; 3–4 components per observer).

We then low-pass filtered the data to 45 Hz (3^rd^ order Butterworth IIR filter), downsampled to 250 Hz and ICA was performed again, this time to obtain a source representation of the MEG data. This and all other Butterworth filters were applied with two passes, once forward, once backward to eliminate phase distortion. ICA is a blind-source separation method that finds fixed spatial patterns (sources) whose activity is temporally maximally independent of activity arising in other sources^47^. Independent sources thus obtained are usually found to be dipolar^48^. We selected the first 60 components ordered by variance explained, low-pass filtered their source time courses with a cutoff of 40 Hz (3^rd^ order Butterworth IIR filter) and down-sampled to 125 Hz (8 ms time bins) for further analyses. We chose 60 components as we found there were between 55 and 60 components for each observer that exhibited some modulation over the experimental time course (measured with the variance over time of the variance over trials). For consistency, we chose 60 for each observer; this is also consistent with the number of dipolar sources obtained from ICA with EEG^48^. The total variance explained by the top 60 components was 85%, 75%, 86%, 81%, 84%, respectively for each observer. We approximated the locations of the ICA sources via a dipole fitting procedure consisting of a coarse grid search followed by a non-linear fit, as implemented in the FieldTrip package^45^. We show approximate source dipole positions in the horizontal plane (axial view) on a schematic brain (Figs 5 and 6), and the full 3d positions of for the functionally identified hub sources in Supplementary Figure S6.

Note that the use of ICA to obtain MEG sources separates the problem of source identification from the problem of source localization. In this application, we wanted to obtain single-trial source time courses, from highly variable single trials and covering a short post-stimulus temporal window of highly dynamic stimulus evoked activity covering a large brain region (i.e. transient flow of visual input from early visual areas). ICA is able to obtain sources by considering not only the transient evoked activity from a source, but using ongoing baseline activity from each source^17^,^48^,^49^,^50^.

We now detail each step of the information processing pipeline and refer the reader to Supplemental Figure S5 for a graphic overview and illustration of each step.

### Behavioral Information Content (What?)

We quantized the pixel values of the bubble masks into 3 equally populated bins, separately for each SF band. The bubble mask consists of scalar values at each pixel representing the visibility of that pixel when the mask is applied. Values lie between 1 (meaning the underlying image is completely visible at that pixel) and 0 (meaning the underlying image is completely blocked at that pixel). We calculated MI between the discrete pixel bins and the observer responses separately for each pixel and SF band. We determined statistical significance from the analytic chi-square null distribution of MI^51^ and Bonferroni corrected across all pixels (64 × 64 pixels × 5 spatial frequencies = 20,480 comparisons). Specifically, we computed *behavioral information images* by contrasting, independently for each pixel in each SF band, “nuns” response trials with “don’t know” response trials (N vs. DK) and “Voltaire” response trials with “don’t know” response trials (V vs. DK). We used a standard direct histogram estimator for MI (3 bins for pixel visibility × 2 responses). These images indicate the regions needed for the respective perceptions (see Fig. 2, Behavioral Information Content, Supplemental Figure S5).

### Brain Information Content (What?)

#### Step 1: MEG Pixel Information Images

For each ICA source and time point in the range 0–600 ms post-stimulus, we calculated MI between bubble masks and MEG source activity, pixel-wise and independently for each SF band. As above, we quantized the bubble mask pixel values into 3 equally populated bins, separately for each SF band. We used a bin-less rank based approach for the MEG activity. That is, we first standardized the MEG activity by estimating its empirical Cumulative Distribution Function (CDF) across trials at each time point, and obtaining for each trial the corresponding value from the inverse CDF of a standard normal (see below). To obtain the empirical CDF for a fixed time point we take the values of the MEG ICA source activation at that time point, across trials. We then rank these values and normalize the ranks to lie between 0 and 1. This gives an estimate of the empirical CDF of the data across trials at that time point. We then estimated MI as:


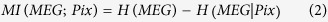


where *H(MEG)* is the entropy of a Gaussian model fit across all trials and *H(MEG|Pix)* is the average of the entropies of Gaussian models fit separately to trials from each pixel bin. Analogous to ANOVA, this approach compares the total variance (the entropy of a Gaussian is a function of the variance) across all trials, to the average within-class variance of the responses to each binned pixel value. We estimated entropy terms and corrected for the bias due to limited sampling using the analytic expressions for Gaussian variables^52^,^53^. While the mutual information estimation in Eq. (2) is parametric, the initial normalization of the MEG distribution means there are no explicit assumptions about the distribution of the MEG data–the resulting MI estimate is a rank statistic. This resulted in a set of 4500 (60 sources × 75 time points) cross-spatial-frequency *MEG-pixel information images*, one for each source and time point.

#### Step 2: Stimulus Features via Dimensionality Reduction

For each observer, we applied Non-negative Matrix Factorization, NMF^18^ to the set of MEG-pixel information images to identify the main stimulus features that modulate MEG source activity. NMF factorizes a high-dimensional data set into a small number of non-negative additive components, where the factorization minimizes reconstruction error. We first smoothed the MEG-pixel information images by 2D convolution with a Gaussian with the same SD as the bubble aperture used for each respective SF. We performed NMF with an alternating-least square algorithm, repeating the calculation 30 times with different random initial conditions, selecting the solution with lowest root-mean-square residual. To determine the number of feature components we repeated the NMF procedure for N = 10 to 25 components. We inspected these manually in sequence and chose the number of components such that the addition of further components did not reveal additional contiguous regions of the stimulus space. This resulted in 21–25 components per observer. We correlated the components with the behavioral classification images to classify them as representing behaviorally relevant content or non-behaviorally relevant content (see Fig. 2, Brain Information Content, for illustrative components). We thresholded the NMF components by setting to zero the pixels with low values (<15% of the maximum component value across SFs) and then normalized them (L2-norm). For each trial and for each NMF feature component we spatially filtered (dot product) the bubble mask for that trial with the normalized NMF component. This resulted in a single scalar value quantifying the degree of presence of that component on that trial. We refer to these values as *stimulus features*.

### Algorithmic Brain Network

#### Step 1. MI between Stimulus Features and ICA Source Activity

For each time point and ICA source, we calculated MI between source activity and stimulus features. Here source activity refers to the values for each trial of the amplitude time-series of the ICA source at the considered time point. Here we calculated MI using a bin-less rank based approach based on copulas. Due to its robustness this approach is particularly well suited for MEG data and provides greater statistical power than MI estimates based on binning the MEG signal. The following paragraphs detail our new MI estimate. It can be skipped without loss of continuity.

A copula^54^ is a statistical structure that expresses the relationship between two random variables. The negative entropy of a copula between two variables is equal to their MI^55^. On this basis, we estimated the MI via the entropy of a Gaussian copula fit to the empirical copula obtained from the data. While the use of a Gaussian copula does impose a parametric assumption on the form of the interaction between the variables, it does not impose any assumptions on the marginal distributions of each variable; this is important as the stimulus features are non-Gaussian. The Gaussian distribution is the maximum entropy distribution for a given mean and covariance, so the Gaussian copula has higher entropy than any other parametric model which fits those statistics. Since the MI is negative copula entropy other choices of parametric copula model could give higher, but not lower values of mutual information. In practice, we calculated the empirical CDF value for each point by ranking the data then scaling the ranks between 0 and 1, and we obtained the corresponding standardized value from the inverse CDF of a standard normal distribution. We computed MI between these standardized variables using the analytic expressions for the entropy of uni- and bi-variate Gaussian variables^52^,^53^. This procedure depends only on the ranks of the input data; therefore our copula MI estimate is a robust lower bound to the MI between two continuous variables.

We determined statistical significance with a permutation approach, and addressed the problem of multiple comparisons using the maximum statistic method^56^. For each of 200 permutations, we randomly shuffled stimulus feature values across trials and repeated the MI calculation for each ICA source and time point. We computed the maximum of the resulting 3D MI matrix (time vs. ICA sources vs. stimulus features) for each permutation and used the 99^th^ percentile of this value across permutations as the statistical threshold.

#### Step 2. Directed Information

*Directed Information* (DI)^57^ from a signal *X* to a signal *Y* quantifies the reduction in uncertainty about the value of *Y* gained from observation of past values of *X*, over and above that which could be obtained from considering the past values of *Y* itself. DI, the most general measure of directed causal functional connectivity^26^, is related to the Wiener^58^ and Granger^59^ principle that interprets an increase in the predictability of signal *Y* from the past of signal *X* as a causal effect of X on Y^60^. The time-resolved version of DI that we use here is equivalent to Transfer Entropy^61^,^62^.

We calculate time-resolved DI as follows. For a pair of ICA sources *X* and *Y*, and a transfer delay *d*, we consider the distribution over trials of the MEG activity of source *Y* at (post-stimulus) time *t* (*Y*_*t*_) and the distributions over trials of the activity of sources *X* and *Y* at time *t – d* (*X*_*t*−*d*_, *Y*_*t*−*d*_). The DI from *X* to *Y* is then equivalent to the *Conditional Mutual Information*^16^ (CMI) between *Y*_*t*_ and *X*_*t*−*d*_ conditioned on *Y*_*t*−*d*_: I(*Y*_*t*_; *X*_*t*−*d*_|*Y*_*t*−*d*_). CMI is the information theoretic analogue of *partial correlation*, and measures the expected value of the information between *Y*_*t*_ and *X*_*t*−*d*_ given *Y*_*t*−*d*_.

Here, we calculate DI using again a Gaussian copula model. First, we normalized each variable across trials by calculating the standard normal value corresponding to the empirical CDF of the value on each trial. Then, we calculated the CMI using the analytic expressions for the entropy of bi- and tri-variate Gaussians^52^,^53^. For each pair of sources we calculated DI independently for each post-stimulus time point and for a range of possible delays.

#### Step 3. Directed Feature Information

To determine the content of the communication quantified by DI, we proceeded as follows. We computed the conditional DI, denoted *DI|F* – the expected value of the DI conditioned on the value of a particular stimulus feature, *F*. The conditioning operation averages (with an integral) *DI* obtained over all specific values of *F*; therefore *DI|F* quantifies the amount of new information transferred from *X* to *Y* that is not related to the variations in the stimulus feature. We then define a novel quantity, *Directed Feature Information* (DFI) as the difference between *DI* and *DI|F*:


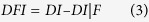


DFI quantifies the causal transfer that is related to the variation of the stimulus feature *F*. If DFI is positive (*DI|F* is less than *DI*, so transfer is reduced on average at fixed stimulus), then it quantifies the amount of DI that relates to variations in the stimulus feature – i.e. is *about* the stimulus feature considered. The above intuition is confirmed by manipulating the expression for DFI using standard information theoretic equalities to obtain:


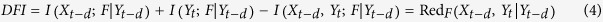


where *Red*_*F*_ (*X*_*t*−*d*_, *Y*_*t*_ *| Y*_*t*–*d*_) represents the so called redundancy^63^,^64^ between *X*_*t*−*d*_ and *Y*_*t*_ about stimulus feature *F* conditional on *Y*_*t*−*d*_. In general, positive redundancy measures the overlap of the MI about the stimulus feature *F* that is present in two different responses. Negative values of redundancy indicate synergy, which occurs when the two variables carry more MI when considered together (because the stimulus modulates their relationship). Therefore positive values of DFI quantify the MI about stimulus feature F that is common to both *X*_*t*−*d*_ and *Y*_*t*_, above and beyond that which would be available in *Y*_*t*−*d*_. Eq. (4) is crucial to allow the interpretation of positive DFI as a measure of transfer of new specific information content from node X to node Y, and we focus on such positive values.

However, it must be noted that the integral that computes redundancy can combine regions with positive values (indicating redundancy) or negative values (indicating synergy)^63^,^65^. Thus, the redundancy we measure is a lower bound on the total redundancy in the system.

#### Step 4. Algorithmic Brain Networks

We computed the time-resolved DI between each directed pair of ICA sources and for delays between 8 and 240 ms (8 ms steps). This large range of delays was chosen to include all possible communication for an overall comparison of networks activated by the stimuli and task. Supplemental Figure S7 shows a histogram of the delays for all significant DI (left) and DFI (right; all features) values found (corrected for multiple comparisons over all pairs of network nodes, time points and delays searched using permutation testing) for Observer 1. To reduce the dimensionality, and to build a picture of the network structure during early processing of the stimulus, we summed the DI values (over time and delay) representing transfer that is both sent and received between 50 ms and 350 ms post-stimulus onset (Fig. 5). This resulted in an asymmetric weighted connectivity matrix^66^. To threshold this matrix we determined significance using permutation testing and the method of maximum statistics^56^. We repeated the DI calculation for 200 permutations in which the trials of the sending source were shuffled with respect to the trials of the receiving source. The 50–350 ms DI was again summed to produce a connectivity matrix and we used as a threshold the 99^th^ percentile of the maximum of this measure over all directed pairs. This procedure results in a family-wise error rate (FWER) of 0.01. We removed sources without a significant connection to any other source for visualization.

Similar to the procedure described above, we computed, for each stimulus feature, the time-resolved DFI between each directed pair of ICA sources and for delays between 8 and 240 ms. We summed the obtained DFI values sent and received between 50–350 ms post-stimulus onset, considering only positive DFI (i.e. transfer *about* the stimulus feature). We determined significant connections using permutation testing and the method of maximum statistics as above; on each permutation the values of the stimulus feature were shuffled across trials.

The above procedure resulted in one weighted asymmetric connectivity matrix per stimulus feature. To investigate properties of the network as a whole we calculated the *total feature degree* of each node^19^,^67^–i.e. the total degree (total number of inward and outward connections) of the node summed across all DFI networks computed from stimulus features that overlap with the behavioral information image. This gives a measure of the network connectivity of the node in terms of transfer of specific behaviorally relevant stimulus features. We identified the node (or nodes) with the highest value of total feature degree as hubs (Fig. 5, greyscale histogram).

For visualization, sources in the displayed connectivity matrices are ordered by total feature degree. We derived the perception-specific algorithmic networks of Fig. 4 by summing the connectivity from DFI networks for stimulus features unambiguously overlapping the respective behavioral information images (this was determined manually by visual comparison between the components and MI images). For example the set of “Nuns” features included the nuns’ faces from the highest SF (red and blue stimulus features in Fig. 5), and the “Voltaire” set included the green stimulus feature for Voltaire.

To investigate the temporal dynamics of the information representation in the identified hubs, we focused on the main behaviorally relevant stimulus features (Red, Green and Blue features from Fig. 5). We considered separately the strongest inward and outward connections to and from the hubs for these stimulus feature networks. These are shown in the left and right schematic figures respectively. Pie charts indicate the maximum feature coding at the nodes (area is scaled according). Several connections are chosen to illustrate the full dynamics of the coding, particularly the relationship between the individual feature representation at the nodes, and the timescale of the peak feature transfer (source time indicated with open circle, arrival time indicated with solid arrowhead).

To determine the statistical significance of the apparent similarity of the functionally identified “hub” nodes (ICA sources, see Supplemental Figure S6) we performed the following analysis. We computed the correlation across sensors of the ICA topologies from different observers for hub sources in the same hemisphere (7 pairs). We then randomly sampled 10,000 pairs of ICA sources from different observers (samples where both sampled sources were hubs were excluded) and calculated the correlation between those topologies. For each of the 7 hub-hub correlations we computed the corresponding percentile of the permutation correlation distribution. The median value of these was 98.93% (range 89–99.92%). A rank sum test showed a significant difference in the median between the hub-hub correlations and the permutation set (p = 1.4 × 10^−5^).

### Network Source Simulation (Fig. 1A)

We simulated baseline activity at two sources with resting state MEG data (3000 trials; 400 ms; 500 Hz sampling rate). For each simulated trial we randomly sampled a contiguous 400 ms segment from 8 minutes of continuously recorded resting state MEG data (single channel). We sampled stimulus feature values across trials from a uniform distribution. On each trial, we added stimulus-evoked activity at source A by linear summation of an activation proportional to the stimulus feature value (Gaussian temporal profile with peak at 124 ms). We modulated source B by adding an activation (Gaussian temporal profile with peak at 224 ms) proportional to the activity of source A in the same trial. The amplitude of this activation was obtained as a weighted sum (Gaussian temporal profile with peak at 124 ms) of the activity at A (including the stimulus modulation). Each simulated trial was low-pass filtered at 30 Hz. We chose these parameters to match the properties of the experimental MEG data set.

We performed analysis as for the experimental data (above), computing MI between feature values and simulated responses independently at each time point for sources A and B. We calculated DI and DFI independently for each time point and for a range of delays (plotted time axis is aligned to receiving source B).

### Network Source Simulation (Supplemental Figure S1)

We simulated an example to demonstrate that DFI can dissociate different underlying communication processes, when both A and B convey MI about F and there is DI between them. We sampled baseline source activity from resting state data and simulated a stimulus feature dependent activation as above. On each trial, if the stimulus feature had a value between 0 and 0.5 we added stimulus-evoked activity at source A by linear summation of an activation proportional to the stimulus feature value (Gaussian temporal profile with peak at 124 ms). If the stimulus feature had a value between 0.5 and 1 we added stimulus-evoked activity at source B (224 ms). In this example, the only information that A transmitted to B was “internal state” information about fluctuations in the ongoing activity of A prior to receiving the stimulus drive. We achieved this by modulating activity at source B by adding, on each trial, an activation (Gaussian temporal profile with peak at 224 ms) proportional to the baseline activity of source A on that trial, before adding the stimulus modulation (weighted sum within a Gaussian temporal profile with peak at 124 ms).

We performed analysis as for the experimental data (above), computing MI between feature values and simulated responses independently at each time point for sources A and B. We calculated DI and DFI independently for each time point and for a range of delays (plotted time axis is aligned to receiving source B).

## Additional Information

**How to cite this article**: Ince, R. A. A. *et al.* Tracing the Flow of Perceptual Features in an Algorithmic Brain Network. *Sci. Rep.* **5**, 17681; doi: 10.1038/srep17681 (2015).

## Supplementary Material

Supplementary Information

## Figures and Tables

**Table 1:** Number of trials after MEG preprocessing.

Observer	All responses	‘Nuns’ response	‘Voltaire’ response	‘Don’t Know’ response
1	3314	1189	1313	812
2	3604	1666	1263	675
3	4145	1634	1892	628
4	3023	1603	738	682
5	2885	1007	1346	532

**Table 2:** Reaction times in ms. Median (25th; 75th %-ile).

Observer	All responses	‘Nuns’ response	‘Voltaire’ response	‘Don’t Know’ response
1	526 (452; 637)	525 (455; 636)	490 (420; 589)	596 (514; 709)
2	681 (579; 865)	644 (549; 786)	719 (607; 951)	730 (616; 893)
3	766 (661; 901)	765 (663; 898)	749 (648; 882)	811 (700; 956)
4	701 (589; 907)	654 (553; 835)	712 (611; 910)	797 (679; 1023)
5	926 (732; 1359)	946 (748; 1312)	816 (680; 1061)	1510 (996; 2424)
