# Supplementary material for: Tracing the Flow of Perceptual Features in an Algorithmic Brain Network
Source: Sci Rep. 2015 Dec 4;5:17681. doi: 10.1038/srep17681 (PMC4669501; doi:10.1038/srep17681)
Supplement: Supplementary Information [file srep17681-s1.pdf]

Supplementary Information For:

Tracing the Flow of Perceptual Features in an Algorithmic Brain Network

Robin A. A. Ince[1], Nicola J. van Rijsbergen[1], Gregor Thut[1], Guillaume A. Rousselet[1], Joachim Gross[1], Stefano Panzeri[1,2] and Philippe G. Schyns[1*]

[1] Institute of Neuroscience and Psychology, University of Glasgow, Glasgow, G12 8QB, UK
[2] Neural Computation Laboratory, Istituto Italiano di Tecnologia, Rovereto, 38068, Italy

[*] Corresponding author: philippe.schyns@glasgow.ac.uk

Supplementary Figure Legends

Supplementary Figures S1-S4. *Revealing algorithmic brain networks from MEG data during a perceptual decision*. Mirroring the structure of Figures 2, 4 and 5, the results of the same analyses are presented for the 4 other observers. The section of the Dali painting "Slave Market with a Disappearing Bust of Voltaire" is © Salvador Dalí, Fundació Gala-Salvador Dalí, VEGAP, 2014 and is excluded from the Creative Commons license covering the rest of this work.

Supplementary Figure S5. *Details of the analysis pipeline*. The *Stimulus sampling* box shows example single trial bubble masks. The *Behavioral Information Content* box shows example data for a single pixel Mutual Information calculation, to illustrate the construction of the behavioral information images. The *Brain Information Content* box illustrates the analysis of MEG data and the process used to obtain stimulus features. The *Algorithmic Brain Network* box illustrates the DI and DFI calculations between a specific pair of sources that we used to build each network link. The section of the Dali painting "Slave Market with a Disappearing Bust of Voltaire" is © Salvador Dalí, Fundació Gala-Salvador Dalí, VEGAP, 2014 and is excluded from the Creative Commons license covering the rest of this work.

Supplementary Figure S6. *ICA Source Topologies and Fitted Dipole Locations*. For each observer, we show topologies of the ICA sources of each of the functionally identified hub network nodes (the input to the dipole fitting algorithm) together with the 3d location (plotted on the individual normalized anatomical MRI) of the fitted dipoles.

Supplementary Figure S7. *Significant communication delays*. Histograms show the delays of all significant DI (left) and DFI (right; all features) connections for Observer 1. Significance (p=0.01) is determined by permutation testing and the method of maximum statistics, which corrects for multiple comparisons over all pairs of network nodes, time points and possible delays.

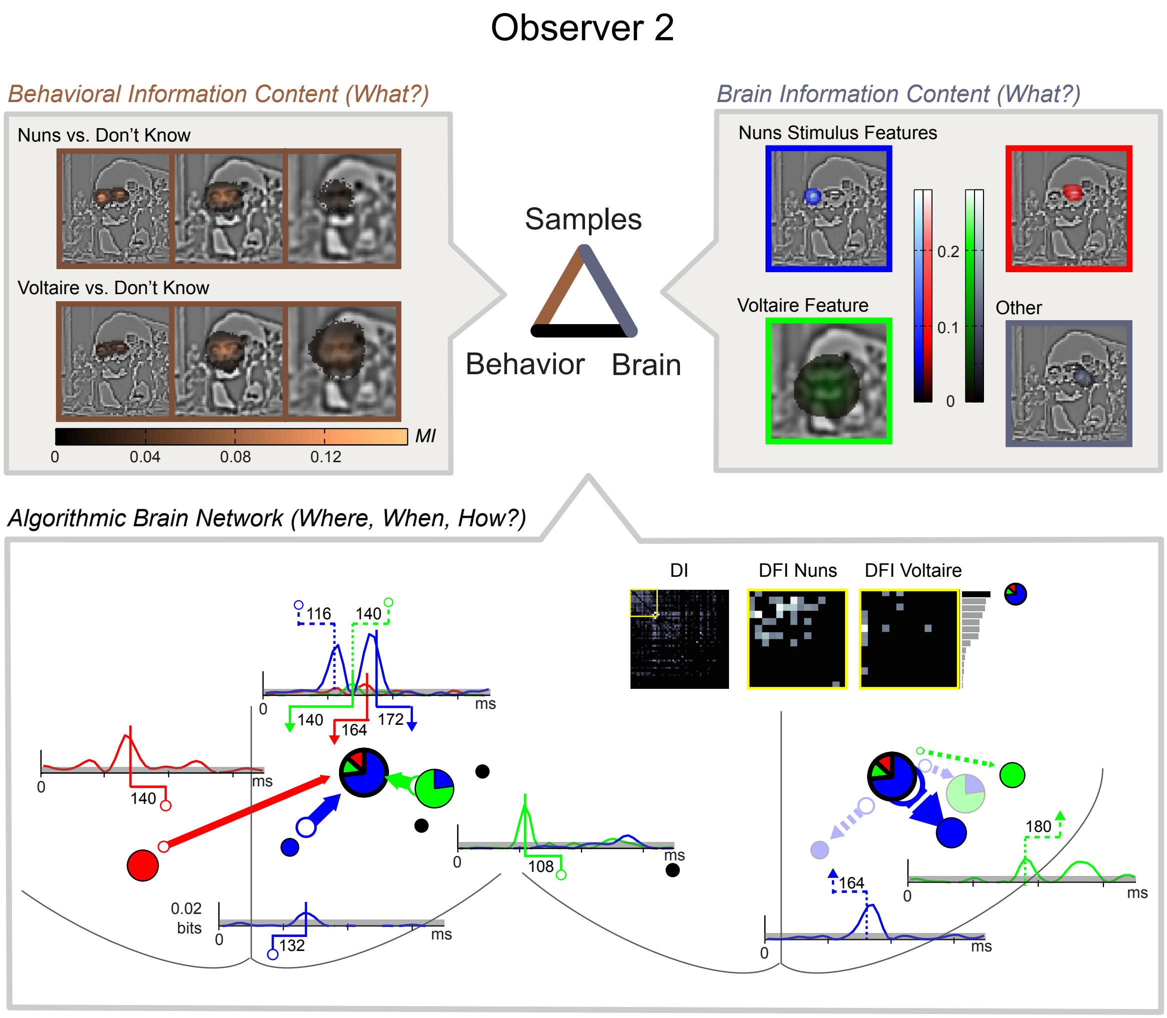

Supp Figure S1

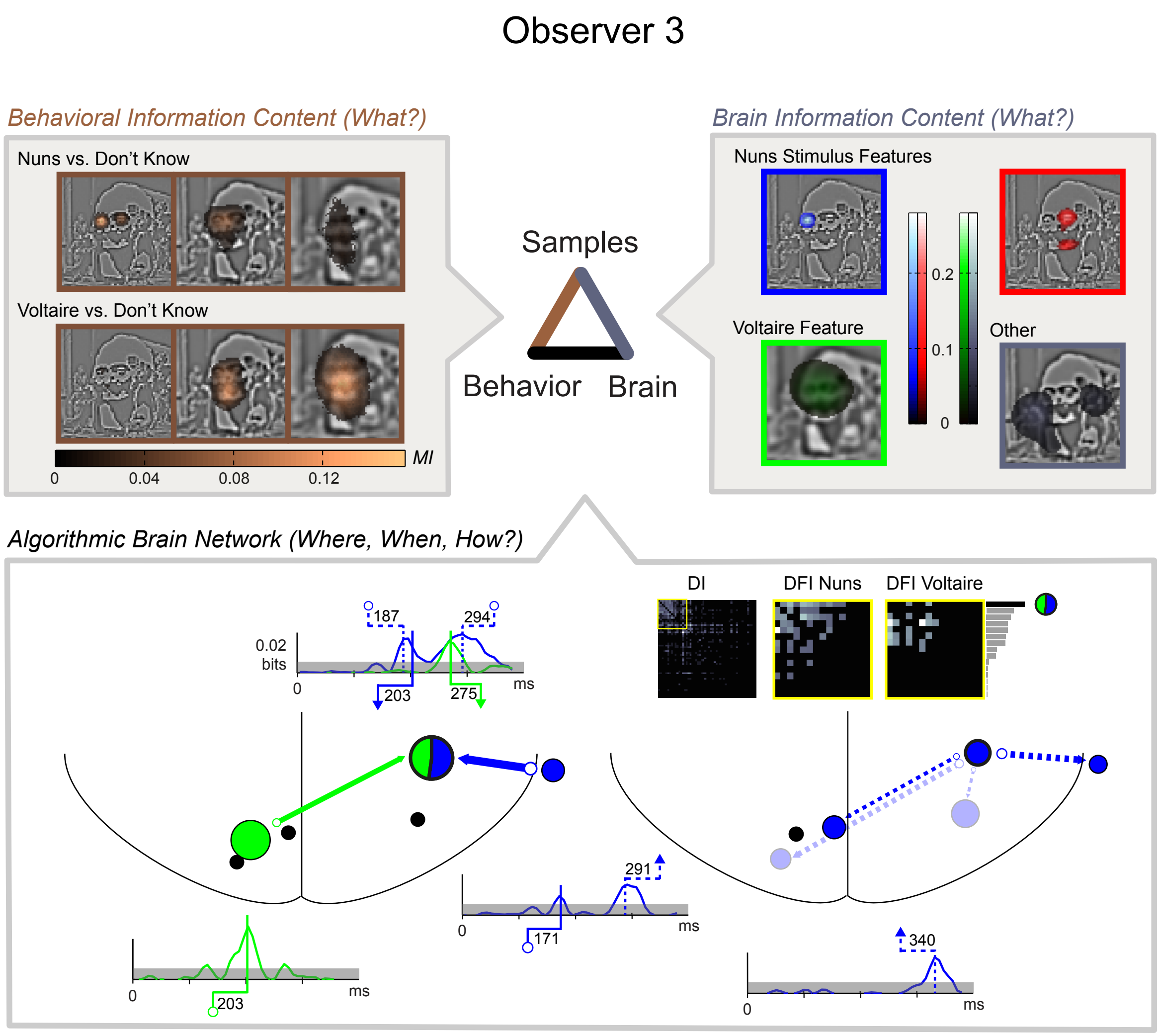

Supp Figure S2

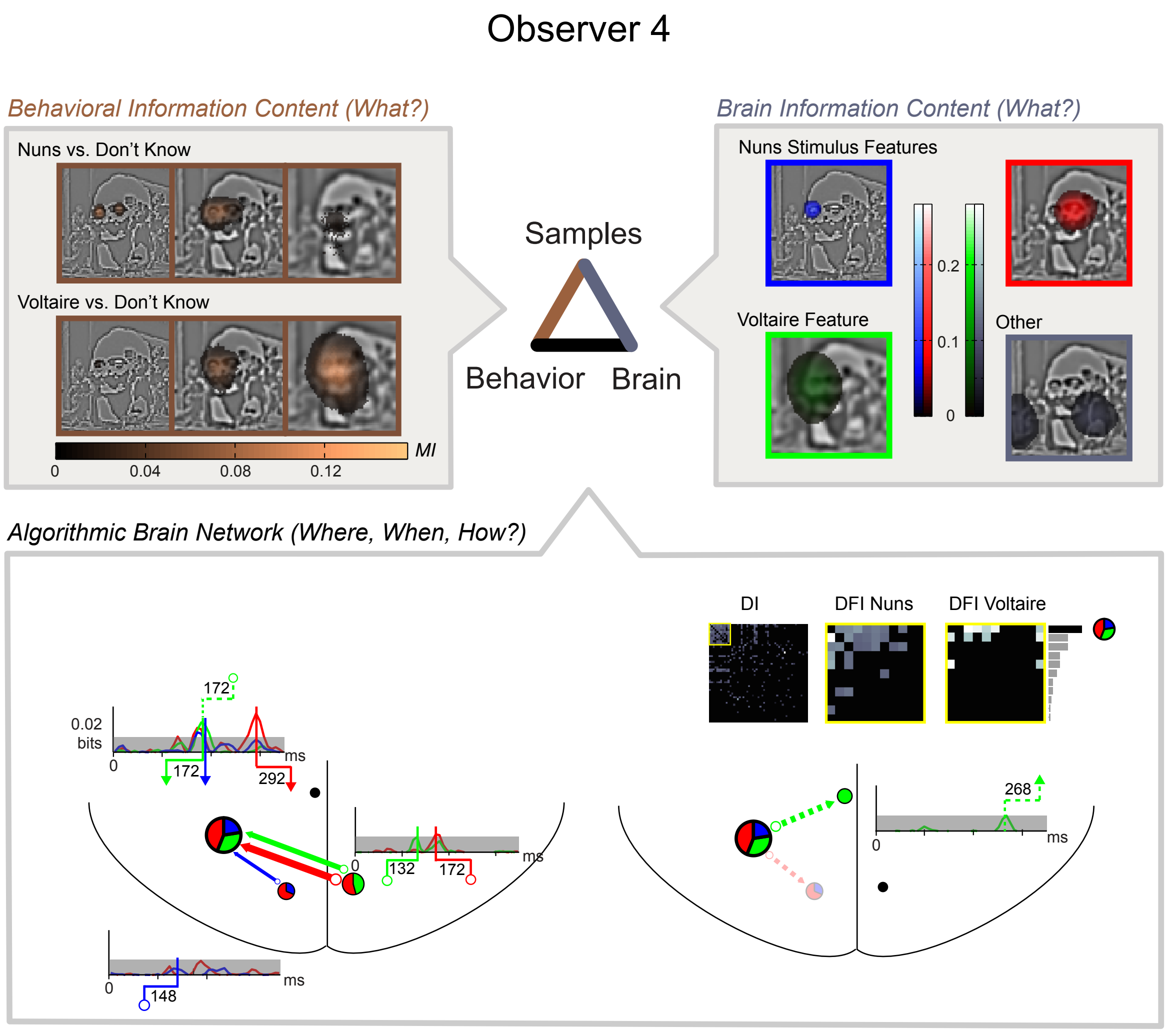

Supp Figure S3

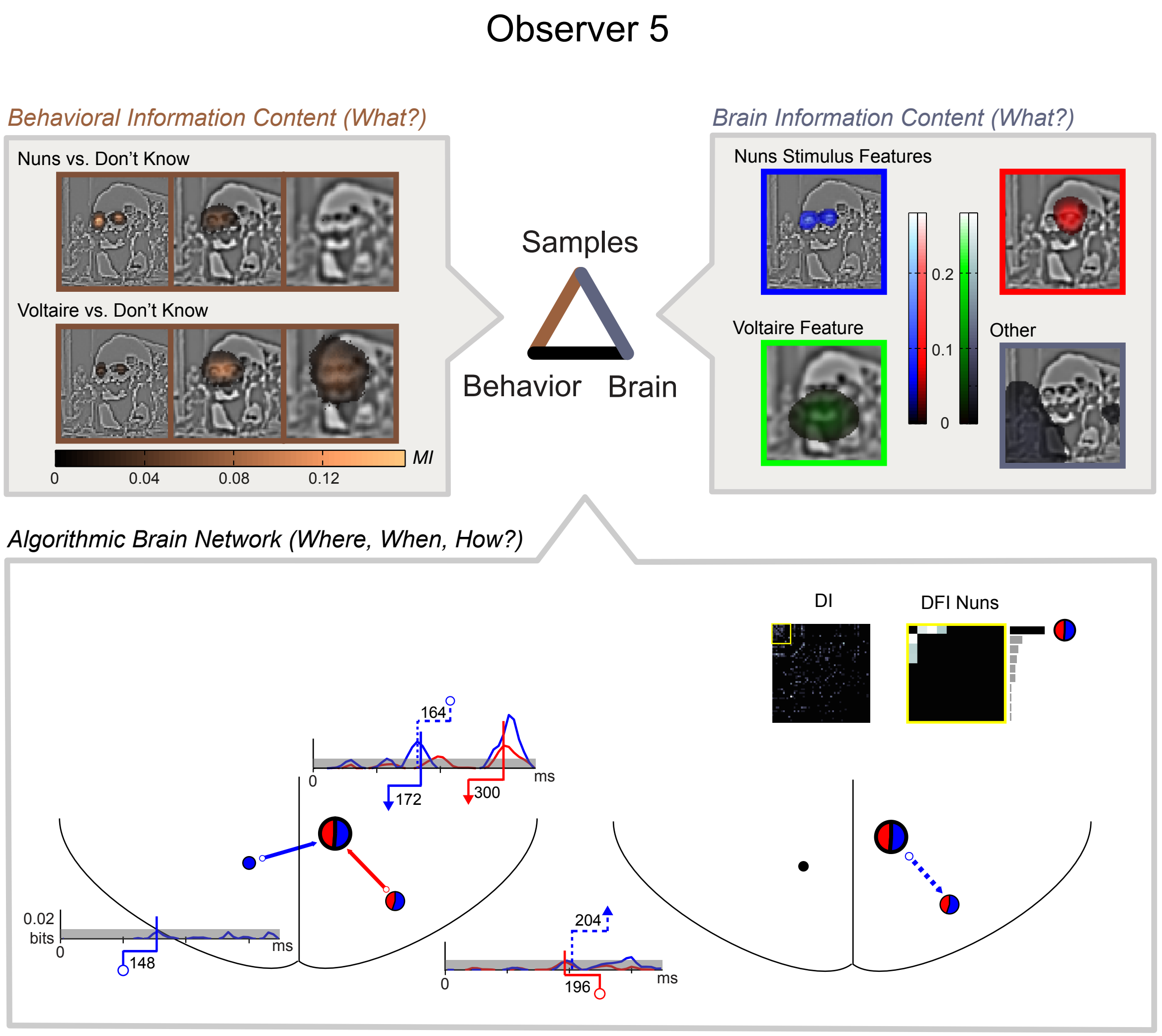

Supp Figure S4

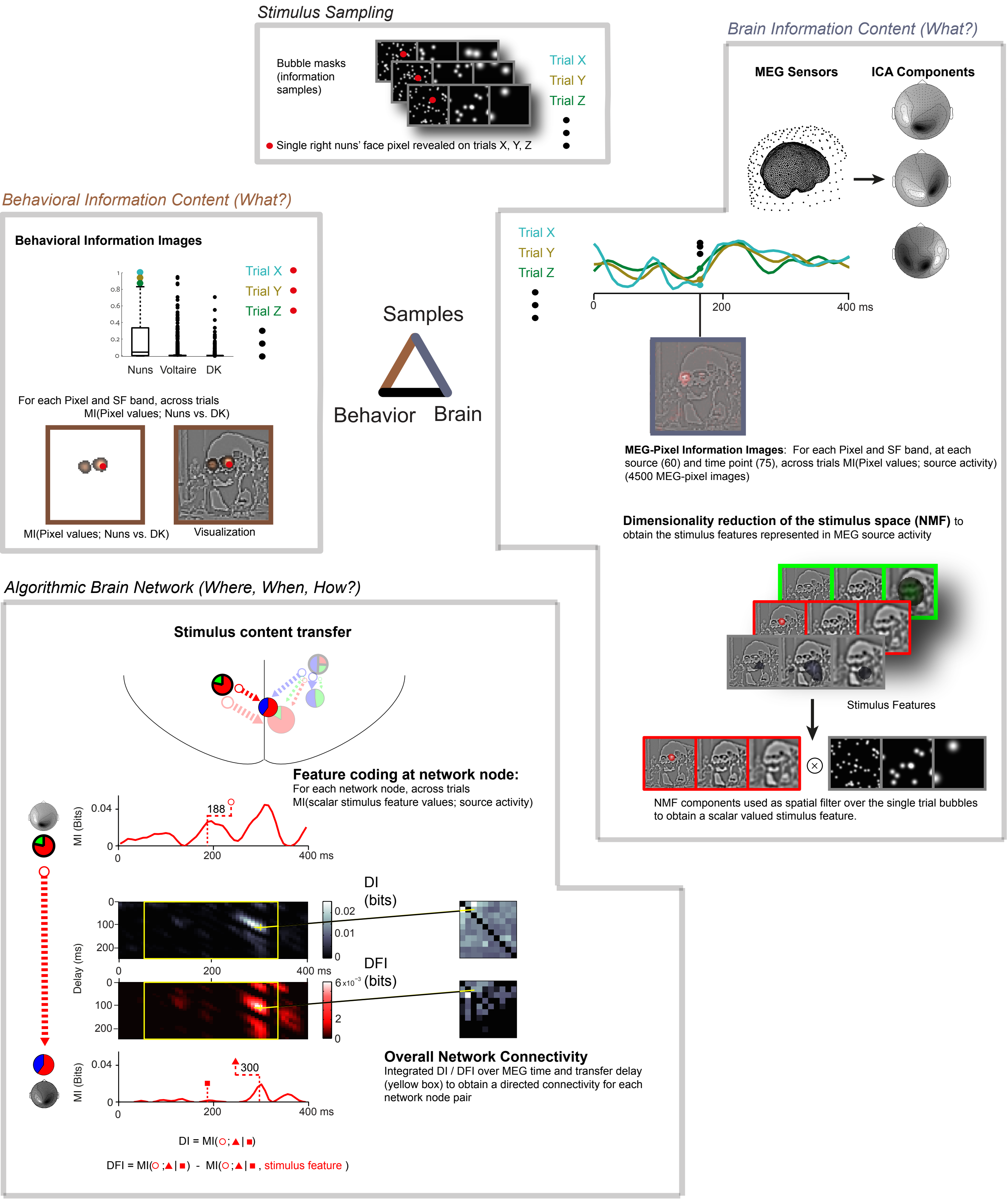

Supp Figure S5

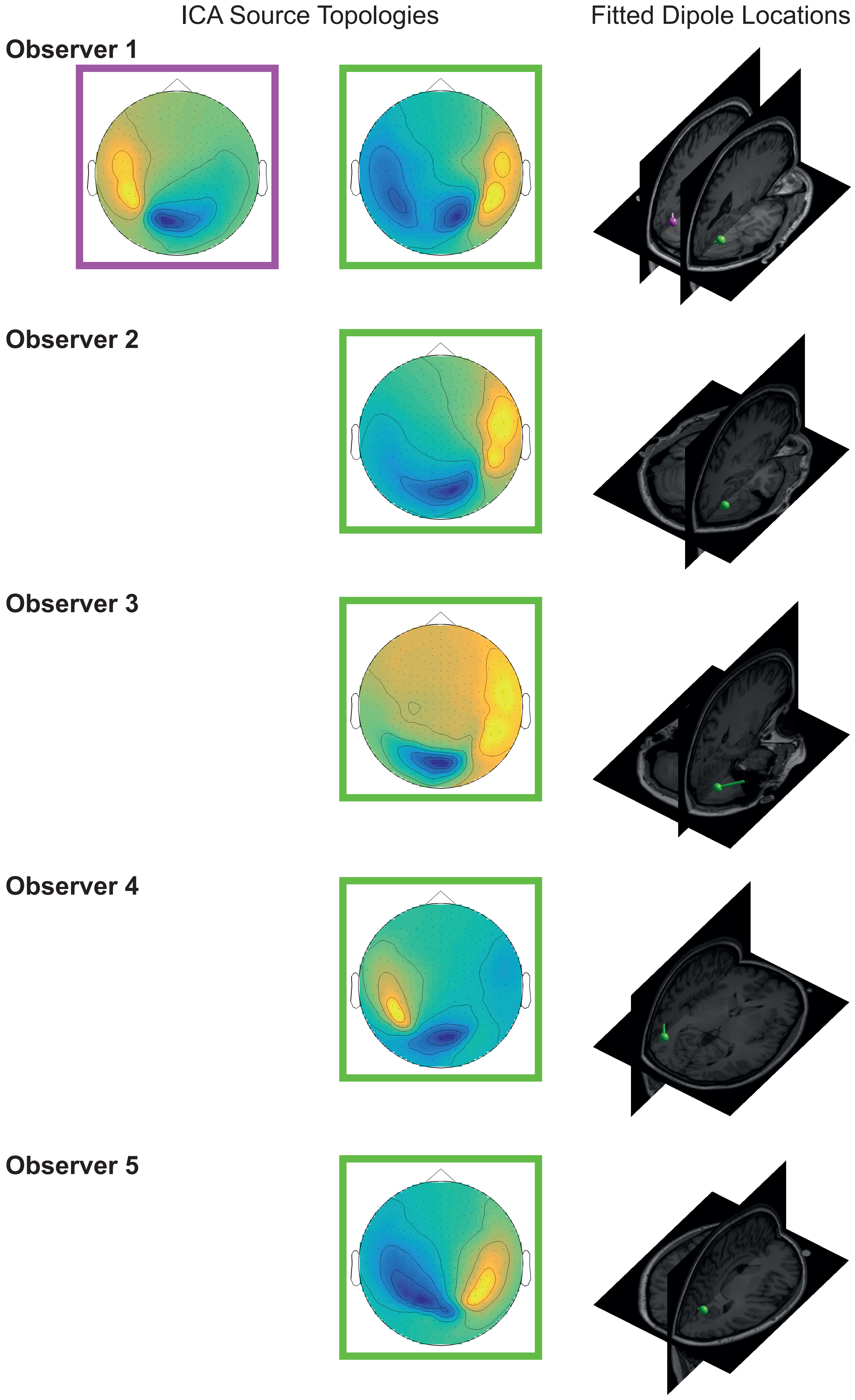

Supp Figure S6

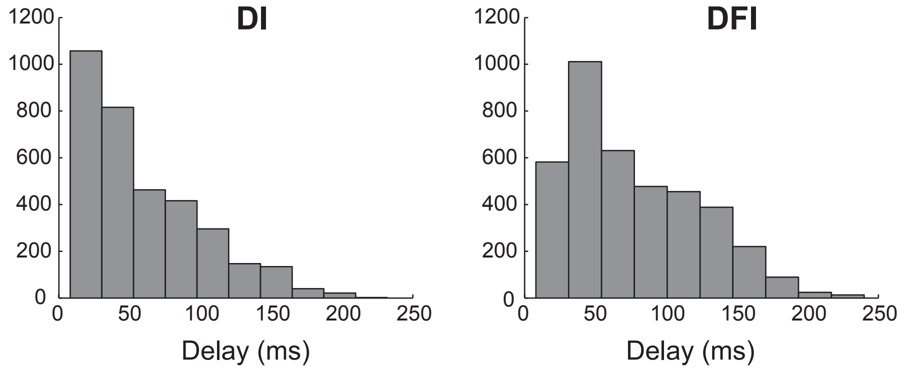

Supp Figure S7
